# Neonatal Group B Streptococcal Disease in Otherwise Healthy Infants: Failure of Specific Neonatal Immune Responses

**DOI:** 10.3389/fimmu.2017.00215

**Published:** 2017-03-07

**Authors:** Alessandro Borghesi, Mauro Stronati, Jacques Fellay

**Affiliations:** ^1^School of Life Sciences, École Polytechnique Fédérale de Lausanne, Lausanne, Switzerland; ^2^Swiss Institute of Bioinformatics, Lausanne, Switzerland; ^3^Neonatal Intensive Care Unit, San Matteo Hospital, Pavia, Italy

**Keywords:** newborn infant, life-threatening, primary immunodeficiency, genetic predisposition to disease, Mendelian diseases, monogenic, infection, group B streptococcus

## Abstract

Only a small proportion of newborn infants exposed to a pathogenic microorganism develop overt infection. Susceptibility to infection in preterm infants and infants with known comorbidities has a likely multifactorial origin and can be often attributed to the concurrence of iatrogenic factors, environmental determinants, underlying pathogenic processes, and probably genetic predisposition. Conversely, infection occurring in otherwise healthy full-term newborn infants is unexplained in most cases. Microbial virulence factors and the unique characteristics of the neonatal immune system only partially account for the interindividual variability in the neonatal immune responses to pathogens. We here suggest that neonatal infection occurring in otherwise healthy infants is caused by a failure of the specific protective immunity to the microorganism. To explain infection in term and preterm infants, we propose an extension of the previously proposed model of the genetic architecture of infectious diseases in humans. We then focus on group B streptococcus (GBS) disease, the best characterized neonatal infection, and outline the potential molecular mechanisms underlying the selective failure of the immune responses against GBS. In light of the recent discoveries of pathogen-specific primary immunodeficiencies and of the role of anticytokine autoantibodies in increasing susceptibility to specific infections, we hypothesize that GBS disease occurring in otherwise healthy infants could reflect an immunodeficiency caused either by rare genetic defects in the infant or by transmitted maternal neutralizing antibodies. These hypotheses are consistent with available epidemiological data, with clinical and epidemiological observations, and with the state of the art of neonatal physiology and disease. Studies should now be designed to comprehensively search for genetic or immunological factors involved in susceptibility to severe neonatal infections.

## Introduction

Neonates are commonly thought to be vulnerable to pathogens because of neonatal immaturity, immune tolerance, or immune deviation, a developmentally regulated transitional state (1–4). These concepts, while useful to describe the highest incidence of infection during the neonatal age at the population level, do not take into account interindividual variability. Even if the highest incidence of infection is observed during the first 28 days of life, the majority of newborn infants are resistant to common pathogens, and only a small proportion of infants exposed to a given microorganism develop overt disease.

It is very clear from epidemiological studies that multiple risk factors contribute to the individual risk of developing neonatal infections. Based on them, neonates can be classified into high- and low-risk groups; individual risks can be estimated; and preventive protocols can be designed for infants who are at high risk of suffering from severe infections (5–7).

Despite their proven clinical utility, most preventive protocols are only partially effective. This can be explained in part by incomplete adherence by healthcare practitioners and missed opportunities (8–10). However, another critical limitation is the inability of current protocols to accurately predict susceptibility to severe infection at the individual level. Furthermore, infections that occur in the absence of any recognizable factors are currently unpredictable.

Many research groups are focusing on the mechanisms of host susceptibility and resistance to pediatric and adult infections (11, 12). Conversely, neonatal infections have been much less studied from a host susceptibility perspective. Several layers of complexity have indeed prevented researchers from fully understanding the neonatal-specific protective immunity, beyond maternal protection of the neonate through transplancentally transmitted antibodies (Abs). Considering the global burden of neonatal infectious diseases, this looks like a missed opportunity to address a critical public health problem.

The study of neonatal infections raises several practical and ethical issues and is challenging from a scientific perspective. First, the neonatal immune system is a rapidly evolving entity, as is every other organ and system soon after birth (13). Second, and possibly more importantly, there is a complex immune interplay between the mother and the child. The maternal environment (the maternal immune system and microbiome) is intertwined with physiological and pathological processes occurring in the fetal and neonatal tissues (e.g., the maturation of fetal and neonatal immune responses, the composition of the neonatal microbiome) (13–17).

To date, little is known about the mechanisms leading to individual vulnerability and resistance to specific pathogens in the neonatal age. We here propose novel, testable hypotheses that could explain the interindividual differences in pathogen susceptibility and help dissect the molecular and cellular bases of severe neonatal infections.

## Epidemiology of Neonatal Infections

The *Global Burden of Disease Study 2015* reports that “sepsis and other neonatal infections” account for 336,300 neonatal deaths each year worldwide (18).

The distribution of infecting microorganisms varies between term and preterm infants and is different in the neonatal period compared to other age groups. Early-onset and late-onset infections are defined as infection occurring during and after the first 6 days of life, respectively. According to other definitions, 48–96 h of life could be used as cutoff (19).

Group B streptococcus (GBS), or *Streptococcus agalactiae*, is one of the leading pathogens in neonatal infections occurring in full-term newborn infants during the first week of life (9). It is also the most frequent cause of sepsis and meningitis in young infants after the first week of life (20, 21). Recent reports show an increase in the proportion of *Escherichia coli* infection, mostly associated with urinary tract infection, in previously healthy, full-term infants aged 1 week to 3 months (22, 23). Other pathogens responsible for invasive infection in full-term infants include Gram-positive (*Staphylococcus aureus, Streptococcus* spp., *Enterococcus* spp., and, less frequently, *Listeria monocytogenes*) and Gram-negative microorganisms (*Klebsiella* spp., *Citrobacter* spp., *Serratia marcescens, Salmonella* spp., *Haemophilus influenzae*) (22, 23). Deep organ infections by *Candida* spp. and other fungal microorganisms are exceedingly rare in full-term infants.

In very low-birth-weight (VLBW; <1,500 g at birth) infants, Gram-negative pathogens are the most frequently isolated microorganism in early-onset infections, while Gram-positive bacteria are the most frequently isolated pathogens in late-onset infections in the neonatal intensive care units (NICUs), followed by Gram-negative bacteria and fungal organisms (24–29).

## Susceptibility to Neonatal Infections

### Maturation of the Neonatal Immune System

The neonatal immune responses differ in many aspects from immune responses in other age groups. A fine-tuning is required to balance the need for tolerance to beneficial antigens (microbial flora and nutrients) and the need for defense against harmful microorganisms.

The cord blood is enriched in CD4+CD25+ T regulatory cells with potent suppressor activity (30, 31) and other immunosuppressive cell populations including some B cell populations and CD71+ erythroid cells (32, 33).

Despite this strong immunosuppressive component, the neonatal immune system has been demonstrated to be able to mount pro-inflammatory responses that are appropriate for the protection against common pathogens in most infants (34). The two main components of the adaptive immune system, the T and B cell compartments, undergo maturation during human fetal life, with progressive and regulated acquisition of B and T cell repertoire diversity and complexity (35). In addition, the human cord blood possesses several pro-inflammatory cell populations, including newborn-specific interleukin (IL)8-producing T cells (36) and a population of CD4+ T cells with a memory-like phenotype and a variety of effector functions (37).

Cells of the neonatal adaptive immune system are capable of mounting a wide range of responses, from poor or “deviant” T helper 2 (Th2)-skewed antiinflammatory responses to balanced Th1/Th2 responses, and even strong adult-like pro-inflammatory responses (2–4, 38). A series of elegant experiments have shown that neonatal T cells, unlike adult cells, are able to produce large amounts of the Th2 cytokines, IL4 and IL13, upon polyclonal stimulation (39, 40). This phenomenon is linked to extensive epigenetic modifications at the Th2 locus (*IL5, IL13*, and *IL4* genes) and in particular to hypomethylation of the conserved non-coding sequence 1 locus, an enhancer and coordinate regulator of Th2 cytokine production (38, 41). Despite this Th2 bias, neonatal adaptive immune responses can shift toward a dominant Th1 and pro-inflammatory response depending on the type of innate responses and the conditions of antigen exposure (38, 42).

Adaptive immune responses require, however, several days to take place. Neonates cannot rely on preexistent immunological memory because exposure to foreign antigens and pathogens is limited during intrauterine life (34, 42). Furthermore, humoral immunity largely depends on maternally transmitted antimicrobial IgG Abs during the first days of life. The rapid decline of maternal IgG in the neonatal plasma after birth (with a half-life of 21–30 days) is accompanied by a relatively slow maturation of both T-dependent and T-independent B-cell responses throughout the first months of life (13, 43).

Infections occurring in the neonatal period are, by definition, primary infections, and neonates mostly rely on the innate immune responses that provide a first line of defense against invading pathogenic microorganisms (34, 44, 45).

A number of studies demonstrated that the neonatal innate immune responses are characterized by dampened Th1-polarizing and pro-inflammatory responses [low amounts of tumor necrosis factor (TNF) upon toll-like receptor (TLR) stimulation] and by increased production of Th2-polarizing and antiinflammatory cytokines (higher IL6/TNF ratio compared to adult responses) (45–48). Furthermore, decreased phosphorylation of signal transducer and activation of transcription 1 in response to interferon gamma (IFN-γ) (49) and developmental maturation of specific dendritic cell subsets (50) contribute to the neonatal-specific Th2-polarizing innate immunity.

Interestingly, full-term healthy newborn infants do not appear specifically vulnerable to deep infection by microorganisms typically causing disease in immunodeficient patients, most notably *Nocardia* and fungi-like *Aspergillus, Candida, Cryptococcus, Pneumocystis*, and other opportunistic pathogens, suggesting a substantial maturation of the specific antifungal protective responses in most full-term newborn infants.

Altogether, the characterization of the neonatal immune responses over the past two decades has shown profound differences with adult immunity that in part explain the overall increased susceptibility to life-threatening infection of newborn infants. However, little is known so far about the interindividual differences in the immune protection against pathogens in the neonatal age.

### Heritability of Neonatal Sepsis

There is controversy over the heritability of susceptibility to neonatal sepsis. In one study, comparing the concordance of late-onset sepsis in same-sex vs. unlike-sex twin pairs, no evidence was found of a genetic component of susceptibility to late-onset sepsis among VLBW infants (51). Conversely, another study compared sepsis concordance rates between monozygotic and dizygotic twins; the authors found that 49% of the variance in liability to late-onset sepsis could be explained by genetic factors alone and 51% by residual environmental factors (52). Both studies focused on cohorts of very preterm/VLBW infants. No study so far addressed the question of heritability of neonatal sepsis in late-preterm and full-term newborn infants.

The question of the role of the genetic background on neonatal host susceptibility to infection has been addressed by several studies. All published studies, included in a recent meta-analysis, used a candidate gene approach on cohorts of preterm infants (53). One genome-wide association study (GWAS) is ongoing (54). Such studies are useful to investigate the contribution of host genetics in the setting of a likely multifactorial pathogenesis, as it is probably the case for most infections occurring in preterm infants. Different approaches are needed to find the genetic determinants of susceptibility to life-threatening infections occurring in full-term infants with no underlying medical conditions in which susceptibility to infection is largely unexplained.

### Lessons from Inborn Errors of Immunity in Pediatric Infections

Inborn errors of immunity or primary immunodeficiencies (PIDs) are a group of genetic disorders characterized by increased susceptibility to infection. Historically, the so-called conventional PIDs have been the first PIDs described and dissected from a molecular perspective (55). They are typically Mendelian diseases, caused by highly penetrant single-gene defects. They often occur in families or in the presence of consanguinity and are characterized by a profound defect in one or more arms of the immune system leading to susceptibility to recurrent infections by a broad range of microorganisms (56).

Over the past two decades, it has become clear that infectious diseases previously thought to be due to the sole virulence of the pathogen may be the expression of a monogenic disorder underlying a PID. Inborn errors of immunity resulting from single-gene defects have been shown to underlie multiple bacterial infections [myeloid differentiation primary response 88 (MYD88) and interleukin 1 receptor-associated kinase 4 (IRAK4) deficiency], monogenic susceptibility to mycobacterial disease (deficiency of genes in the IL12/IFN-γ loop), herpes simplex encephalitis (defect in TLR3-dependent immune responses), and severe primary *Influenza* virus infection (interferon regulatory factor 7 deficiency) (56–60).

These “non-conventional” PIDs are distinguished from conventional PIDs as they often occur in sporadic cases without any family history of severe infection. Individuals affected by non-conventional PIDs are often otherwise healthy. The immunological phenotype is not detectable with first-line immunological studies, and the disease might manifest as a single episode of severe and potentially lethal infection caused by a common or opportunistic pathogen, mostly during primary infection (56, 58, 59).

The discovery of non-conventional PIDs suggested that monogenic conditions might underlie infectious diseases of infancy and childhood more frequently than previously thought (11). The model of the genetic architecture of human infectious diseases that has been proposed based on these observations suggests that infections occurring early in life are more likely to be caused by single-gene disorders (61).

### PIDs in Neonatal Infections

The proportion of neonatal infections that can be explained by known PIDs is unknown. However, there is evidence from case reports or small case studies that life-threatening infections occurring early in life may represent the first phenotypic manifestation of an inborn error of immunity.

The role of conventional PIDs in conferring susceptibility to infection in the neonatal age has been recently reviewed by Walkovich and Connelly (62). It is important here to remember that a high index of suspicion is required, given that the infectious and potential extraimmunological phenotypes may be only partially expressed during the neonatal period.

Non-conventional PIDs have also been shown to underlie life-threatening neonatal infections. Pyogenic infections occurring during the first few weeks of life have been described as the first phenotypic manifestation of IRAK4 and MYD88 deficiencies (63–65). *Klebsiella pneumoniae* infection often striking in neonatal units as a fulminant and fatal disease, has been linked in some pediatric patients to IL12 receptor subunit beta 1 deficiency (66).

Loss-of-function mutations in interferon induced with helicase c domain 1 (*IFIH1*), a cytosolic sensor of the viral RNA, have been implicated as causative factors in lower respiratory tract infections (pneumonitis, bronchiolitis) caused by RNA viruses (67). Interestingly, the phenotype of IFIH1 deficiency is narrow (restricted to few related RNA viruses), transient (recurrence was found in one of eight patients), and organ specific (only affects the lungs).

Variants in single Ig And TIR domain containing (*SIGIRR*) have been implicated as a possible causative or facilitating factor of necrotizing enterocolitis (NEC) (68), but fulminant and infection-associated NEC (69) in infants with no other identifiable facilitating iatrogenic factor or medical condition has not been linked yet to a genetic condition.

### Spectrum of Neonatal Infections

From a clinical perspective, newborn infants suffering from life-threatening infections may be divided in two major groups:

(1) Newborn infants with a known medical condition. This group includes all infants admitted to a NICU (therefore exposed to nosocomial pathogens) and specifically very preterm (<32 weeks gestational age) and extremely preterm (<28 weeks gestational age) infants, infants undergoing surgery, infants with organ disease (e.g., urinary tract malformations, neurological conditions), and infants receiving medical procedures or treatments that are *per se* sufficient to explain an increased vulnerability to colonizing microorganisms. Infections in this group are multifactorial or linked to one specific known factor of vulnerability, and only a small proportion of the risk is probably explained by individual genetic variation.
(2) Otherwise healthy, full-term, or late-preterm newborn infants with no identifiable medical conditions. Severe infections in these infants occur without any apparent risk or facilitating factor and, from a host perspective, can be considered idiopathic diseases.

Most of these infections occur as isolated events (the spectrum of susceptibility is extremely narrow, in most cases restricted to a single microorganism) and rarely recur.

Some infections are almost never observed in healthy children after the first year of life or in adults. These include neonatal GBS disease, viral bronchiolitis, and rare cases of infection-related NEC in late preterm and full-term infants. Conversely, other infections are not age specific, but may occur with particular frequency and severity in the neonatal period and infancy. These include infections by *E. coli, Klebsiella* spp., *Listeria monocytogenes*, and other Gram-negative and Gram-positive pathogens.

The biological underpinnings of the interindividual differences in resistance and vulnerability to specific pathogens in otherwise healthy infants are currently unknown.

### Hypothetical General Model for Neonatal Infections

A general model to explain susceptibility to neonatal infections in full-term and preterm infants is lacking.

Single factors with high effect size explain some of the most severe diseases occurring in infants without known comorbidities. A prime example in neonatal medicine is the rare occurrence of rapidly progressive neonatal jaundice and kernicterus in otherwise healthy, full-term babies, which is due to neonatal hemolysis resulting from either monogenic defects (e.g., spherocytosis, G6PD deficiency) or alloimmune maternal Abs (anti-Rh, anti-ABO) (70). Conversely, hemolysis leading to kernicterus in extremely preterm infants is more likely to be multifactorial, depending on the combined contribution of common genetic polymorphisms, underlying medical conditions, iatrogenic factors, and other environmental determinants (71).

As a general observation, single-gene or single-factor disorders are more likely to underlie severe neonatal disease phenotypes in otherwise healthy full-term infants, while a multifactorial pathogenesis is more likely to explain mild-to-severe neonatal disease in the presence of comorbidities or iatrogenic factors, with severity depending on the underlying pathogenic process (Table 1).

**Table 1:** **Mechanisms of disease in term and preterm infants**.

Involved tissue/organ	Disease phenotype	Single-factor disorders	Multifactorial conditions
Red cells, liver	Neonatal jaundice with/without bilirubin encephalopathy	**Monogenic disorders** (spherocytosis, elliptocytosis, G6PDH deficiency, Lucey–Driscoll and Crigler–Najjar syndromes)	Prematurity, metabolic or respiratory acidosis, alterations of blood–brain barrier, hypoproteinemia, liver immaturity, polycythemia
		**Maternal Abs** (ABO alloimmunization, Rh alloimmunization)	

Megakaryocytic lineage	Neonatal thrombocytopenia	**Monogenic disorders** (genetic thrombocytopenias)	Mild thrombocytopenia in small-for-gestational-age infants, infants with perinatal asphyxia; thrombocytopenia in infants with bacterial and viral infections and/or intravascular disseminated coagulation
		**Maternal Abs** (auto- or alloimmune thrombocytopenia)	

Thyroid	Neonatal hypothyroidism	**Monogenic disorders** (genetic thyroid dysgenesis and dyshormonogenesis)	Maternal exposure to iodopovidone, iodopovidone use in term and preterm infants (Wolff–Chaikoff effect due to iodine transdermal resorption)
		**Maternal Abs** (anti-TPO, anti-TSHr, anti-TG)	

Immune system	Neonatal infection	Urinary tract malformation Mendelian predisposition to life-threatening infection? Maternal anti-cytokine Abs?	Infections in infants with underlying medical conditions facilitating exposure and translocation of the pathogens to the bloodstream

*This table reports examples of hematological and non-hematological neonatal disease phenotypes that can be explained by either monofactorial or multifactorial conditions. The list is non-comprehensive, and other conditions explained by the same mechanisms include neonatal hyperthyroidism, arrhythmias and neuromuscular disorders. Monofactorial conditions, that include monogenic disorders and pathogenic maternal Abs are, in general, severe, often explain disease in full-term infants but can also underlie disease in preterm infants. Disease phenotypes linked to multifactorial conditions can be mild to severe and are generally found in infants with co-morbidities*.

*Abs, antibodies; GSPDH, glucose-6-phosphate dehydrogenase; TPO, thyroperoxidase; TSHr, thyroid-stimulating hormone receptor; TG, thyreoglobulin*.

Along the same lines, we here suggest that single factors with high effect size may underlie life-threatening infections in otherwise healthy, full-term, or late-preterm babies, while a polygenic/multifactorial model may better explain the occurrence and severity of infections in very and extremely preterm infants.

**Figure 1:**
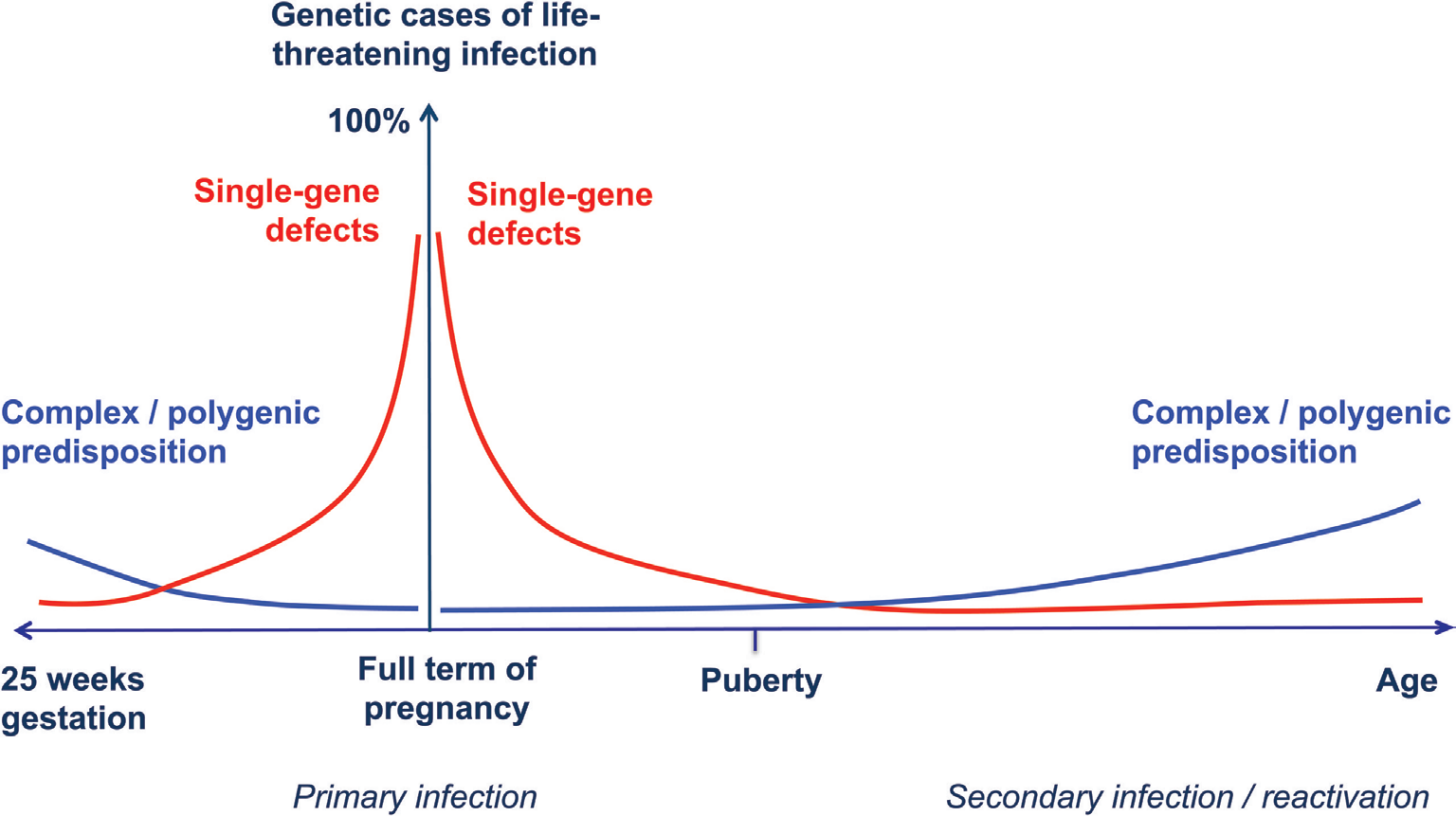
**Human genetic architecture of infections**. Modified from Alcais et al. (61). The contribution of Mendelian genetic defects (red lines) to life-threatening infectious diseases is mostly observed during childhood, while complex interactions between environmental influences and polygenic susceptibility (blue lines) play a more important role for infections occurring later in life. We propose a specular trend for the contribution of human genetic variants to infection susceptibility with decreasing gestational age. In newborn infants at extremely low gestational ages, exogenous factors play a major role, while host genetic defects are more likely to explain life-threatening infection in full-term, otherwise healthy babies.

Accordingly, we propose an extension of the model of the genetic architecture of infectious diseases proposed by Alcais et al. (61) to include full-term and preterm infants (Figure 1).

Additional host factors (maternal antimicrobial protective Abs, vaginal and breast microbiome, epigenetic, and maturational changes in the immune system) and determinants of microbial virulence may also modulate disease severity (17).

### Current Evidence Supporting the Model

In preterm infants, multiple factors are well known to contribute to both the occurrence and the severity of infections. Colonization of deep mucosal tissues by hospital-acquired microorganisms and translocation to the bloodstream is facilitated by several factors: biomedical devices (endotracheal or nasogastric tubes), invasive procedures, thin skin and mucosal layers, central catheters, total parenteral nutrition, drugs (histamine type 2 receptor-antagonists, steroids, antibiotics), delayed initiation of enteral nutrition with formula milk, associated diseases, male gender, an incomplete maturation of the preterm immune system (6, 35, 72–74), and, possibly, a weak polygenic predisposition (52).

In full-term infants, supporting evidence for a role of single host factors in determining susceptibility to infection is provided by the example of urinary tract malformation as one single, high effect-size factor, in determining susceptibility to urosepsis (75) independently of other protective or risk factors. In infants with urinary tract malformation, the effect of the alterations in urinary flow on the facilitation of urosepsis exceeds by far the effect of other potentially modulating factors.

Where no apparent determinant of higher susceptibility to infection is identified, a failure of the individual specific protective innate immune responses can be hypothesized. The failure of specific arms of the immune system that are non-redundant in the neonatal defense against a given microorganism would exceed in effect size the modulating potential of other protective factors.

The view of single-gene defects contributing to the burden of neonatal infections in otherwise healthy infants is supported by the growing body of evidence in the literature describing neonatal infections as the first phenotypic manifestation of a known conventional or non-conventional PID (62–65, 67, 76). However, the great majority of neonatal infections still need to be characterized from a host molecular perspective.

## Neonatal GBS Disease

In the past years, given its predominant role among neonatal infections, neonatal GBS disease has been extensively characterized from an epidemiological standpoint. The elucidation of the mechanisms underlying neonatal vulnerability to GBS may serve as a model to understand the pathogenesis of other neonatal infectious diseases. In the following paragraphs, we discuss the unique susceptibility to GBS infection of some young infants and propose that it could be due to genetic or immune factors.

### Epidemiology and Clinical Characteristics

Group B streptococcus is a Gram-positive, β-hemolytic bacterium frequently colonizing the human gastrointestinal and genitourinary tracts. Invasive GBS disease is extremely rare in healthy adults, with a reported incidence of 10/100,000 non-pregnant individuals (20, 21). Young infants, pregnant and post-partum women, and older adults with underlying medical conditions display higher rates of invasive disease (77).

The global incidence of neonatal GBS disease is estimated to be as high as 0.53/1,000 live births (78). The incidence is highest in infants during the first 3 months of life and dramatically declines afterward (7). Early-onset GBS disease (EOD, onset during the first 6 days of life) occurs after vertical transmission of the bacterium through ascending infection or during delivery through a GBS-colonized birth canal. Risk of EOD can be reduced by administration of antibiotics to the mother during labor. Late-onset GBS disease (LOD) (onset between 7 and 89 days of life) is thought to result from horizontal transmission in most cases. The source of GBS can been identified in some cases. Potential routes of transmission include persistent mucous membrane and skin colonization from acquisition of GBS at birth or after birth from mothers with vaginal colonization; gut colonization through ingestion of infected breast milk from mothers with or without mastitis; or the community or hospital environment (15, 79–81). No preventive strategy exists for LOD. After the introduction of *intrapartum* antibiotic prophylaxis in clinical practice, the incidence of EOD has dropped in the United States from 1.7/1,000 live births in 1993 to ~0.3/1,000 live births, but the incidence of LOD remained stable (7, 82). Clinically, neonatal GBS disease has the features of a severe, life-threatening bacterial infection with systemic disease (sepsis), often associated with organ involvement (meningitis, osteoarthritis, NEC), requiring admission to a NICU. Untreated, it is almost always fatal with multiorgan failure due to septic shock and disseminated intravascular coagulopathy. Case-fatality ratio was as high as 50% in the 1970s (7) and has now dropped to <10% (78, 82), thanks to improvements in neonatal intensive care techniques and the prompt detection of clinical signs of infection and immediate initiation of antibiotic treatment.

### Established Risk Factors for Human Neonatal GBS Disease and Gaps in Knowledge

Approximately 50% of infants born to GBS-colonized mothers (10–30% of all pregnancies) are in turn colonized. Of these, only 1–2% develops overt EOD (7). Data on the proportion of GBS-exposed infants developing LOD are lacking, but it is probably low, given a likely increase in the cumulative exposure/colonization rate with age and a concurrent decline in the incidence of GBS disease.

During the past decades, epidemiological studies led to the identification of several risk factors for EOD, including maternal colonization with GBS and bacteriuria, prematurity, chorioamnionitis, and/or *intrapartum* fever, prolonged (>18 h) premature rupture of membranes (PROM), low maternal anticapsular polysaccharide GBS Abs (29, 83–88), and GBS disease in an older sibling (89). Established risk factors for LOD include prematurity and gut colonization by the pathogen (80, 90).

In many cases, invasive GBS infection develops in otherwise healthy, full-term newborn infants, with as many as 42% of early-onset cases (91) and most late-onset cases occurring in the absence of any established risk factor. Known risk factors are therefore unable to reliably predict the occurrence of GBS disease at the individual level. Rather, they identify groups of infants enriched for determinants of susceptibility, but the nature of such determinants has remained elusive.

### GBS Microbial Load and Virulence Factors

Fetal and neonatal exposure to the microorganism is the *sine qua non-for* neonatal colonization and subsequent infection. Heavy maternal vaginal colonization has since long been recognized as a risk factor for EOD, possibly due to greater bacterial inoculum to the lungs (7). High bacterial load in maternal milk has been linked to neonatal gut colonization and subsequent invasive LOD (80). The determinants of maternal carriage and the maternal bacterial overgrowth are poorly understood. Mild maternal disease may accompany heavy maternal colonization: maternal GBS urinary tract infection in pregnancy is considered a sign of heavy colonization (7), and maternal mastitis may be responsible for high bacterial load in maternal milk (80).

Additional microbial factors, beyond bacterial load, contribute to the development of invasive disease. Ten different GBS serotypes have been described (Ia, Ib, II–IX), based on the capsular polysaccharide antigen. Serotypes Ia, Ib, II, III, and V are most frequently found in EOD; serotype III is the most frequently isolated serotype in LOD and meningitis, but all serotypes can cause neonatal infection (19, 82, 92, 93). The capsular polysaccharide is thought to contribute to the virulence of the microorganism by aiding to escape the host immune responses. Deeper investigation on GBS isolates through multilocus sequence typing and grouping of genetically related sequence types (STs) into clonal complexes (CCs) has shown that most human isolates belong to few CCs (CC1, CC10, CC17, CC19, CC23, and CC26) (94–98) (http://pubmlst.org/sagalactiae/). The hypervirulent CC17 strains (including the hypervirulent ST-17 strain) are newborn specific. They possess the adhesin HvgA and other surface proteins conferring the ability to invade the neonatal central nervous system and are responsible for most LOD with meningitis, but are usually not responsible for adult disease (99). Strains belonging to all the six CCs have been reported in EOD (82, 98, 99).

The neonatal-specific hypervirulence of some bacterial strains and the bacterial load may explain in part the occurrence of neonatal disease. Nonetheless, individual susceptibility is not fully explained by bacterial virulence, especially in cases in which infection is caused by non-hypervirulent strains.

### Protective Immunity to GBS

One fundamental and yet-unanswered question in the field is which are the non-redundant pathways of the innate immune system conferring neonatal protection to GBS.

Several different methodologies in *in vitro* and animal models have been used to attempt to answer this question.

Both knockout mouse and *in vitro* models of GBS infection identified a critical role for TLR and IL1 receptor signaling and/or signaling through MYD88 in bacterial clearance, TNF-mediated inflammation, septic shock, and microglia activation and neurodegeneration (100–109). Specifically, TLR2 and IL1R signaling have been shown to be both beneficial and harmful, depending on the experimental conditions (101, 110–112). A role for IL6, IL10, IL12, and IL18 has been demonstrated in mouse models of GBS infection through administration of anticytokine specific Abs (113–116).

The relevance of the studied pathways in the experimental settings may largely depend on the experimental conditions. Conversely, the non-redundant role of the studied signaling pathways in the human model in natural (as opposed to experimental) conditions still needs to be elucidated (117).

One human study suggested that a null polymorphism in sialic acid-binding immunoglobulin-like lectin 14 (*SIGLEC14*) influences human inflammatory responses to GBS in neutrophils and amniotic membranes and is possibly correlated with GBS-related preterm birth (118), but no data are available on the possible role of SIGLEC proteins in the pathogenesis of GBS infection.

## Hypothesis

Despite advances in the understanding of both the host and the microbial sides of neonatal GBS infection, currently available data are not able to fully explain neonatal susceptibility to infection at the individual level.

We hypothesize that susceptibility to neonatal GBS disease in otherwise healthy infants is due to a failure of the specific neonatal protective innate immune responses to GBS. This neonatal immunodeficiency could be either intrinsic (genetic defect in the infant) or extrinsic/environmental (interference of maternal Abs). In the next paragraphs, we present the genetic and the “maternal antibody” hypotheses of GBS disease and explain how these fit with current evidence.

### The Genetic Hypothesis of GBS Disease

Several reports, recently reviewed (76), demonstrate that adult and neonatal GBS infection may be a phenotypic expression of both conventional (Kostmann disease, transient hypogammaglobulinemia of infancy, chronic granulomatous disease, activated phosphatidylinositol 3-kinase δ syndrome—like immunodeficiency, C2 and IgG4 subclass deficiency, and isolated congenital asplenia) and non-conventional (IRAK4 and MYD88 deficiency) PIDs. Even when occurring in the context of a non-conventional PID, neonatal GBS infection may be one of the several manifestations of a broader phenotype that, for MYD88 and IRAK4 deficiency, includes susceptibility to multiple pyogenic bacteria. Conversely, most cases of neonatal GBS disease occur as an isolated infection, indicating that the susceptibility to GBS is pathogen specific and not linked to a more general state of immunosuppression.

We hypothesize that inborn errors of the primary innate immune responses to GBS, i.e., monogenic susceptibility to GBS disease, underlie some cases of isolated neonatal GBS infection occurring in otherwise healthy neonates. The clinical and immunological phenotypes of isolated neonatal GBS disease may indeed be consistent with those of non-conventional PIDs (57): (i) GBS disease is a potentially lethal infection striking early in life; (ii) the infecting strain/serotype and its virulence factors, while accounting for some variability in the occurrence and severity of infection (92, 119, 120), are not sufficient to explain susceptibility and resistance at the individual level; (iii) the spectrum of susceptibility is extremely narrow, restricted to GBS; and (iv) in most cases, there are no immunological defects at first-line immunological studies that would be consistent with conventional PIDs. In addition, GBS infection usually strikes once in life and only rarely recurs (~1% of cases) (121). This observation is consistent with a low recurrence rate in the subset of non-conventional PIDs characterized by immunodeficiency of the protective immunity to primary infections (57).

The highest incidence of GBS disease during the first 3 months of life would be explained by the high likelihood of being exposed to GBS in the perinatal period and/or by the full penetrance of the genetic defects in this age group.

Recurrence of GBS infection concerns only a small percentage of cases, both singletons and twins, and has been linked to re-exposure to GBS through maternal milk or other sources, to inappropriate treatment, or to persistence of GBS on skin and mucosal surfaces after the first infectious episode (122–125). A genetic explanation for recurrence is also plausible. In some PIDs of protective immunity to primary infection, the genotype has been shown to influence the recurrence rate (126). Therefore, recurrence of neonatal GBS disease may indicate a more severe phenotype or represent the phenotypic manifestation of a specific genetic defect.

The occurrence of GBS disease in siblings (89), as well as the recurrence described in a consanguineous family (76), suggests that the genetic hypothesis may be a plausible explanation for some cases. Infection by poorly virulent strains, the presence of other cases with overlapping phenotypes in the family, consanguinity in the parents, recurrence and severity of the clinical signs, and slow or absent response to antimicrobials despite appropriate treatment strengthen (although their absence does not exclude) the hypothesis of a PID underlying GBS infection.

### The “Maternal Antibody” Hypothesis of GBS Disease

The highest incidence of GBS disease is registered during the first 3 months of life, with most cases (77–78%) occurring during the first week of life (20, 21). This observation, together with a known role of GBS in prenatal disease, both prematurely and at full term of pregnancy (GBS-related stillbirth, term or preterm PROM, chorioaminionitis), suggest that a maternal factor might be particularly important for perinatal infection.

Recently, neutralizing anticytokine auto-Abs have been found in adult and pediatric patients suffering from life-threatening infections, revealing novel mechanisms of unusual susceptibility to specific pathogens (127–130). Auto-Abs against IL17 and/or IL22 have been associated with chronic mucocutaneous candidiasis; anti-IFN-γ auto-Abs with adult-onset immunodeficiency; anti-IL6 auto-Abs with recurrent skin infection; and auto-Abs against GM-CSF with pulmonary alveolar proteinosis (131).

The clinical phenotypes resulting from anticytokine auto-Abs partially (anti-IFN-γ, anti-IL6) or completely (anti-IL17, anti-GM-CSF) overlap with known monogenic conditions affecting the same pathways, demonstrating that Ab-mediated diseases may be immunophenocopies of monogenic immune disorders.

In neonates, autoimmunity is an exceedingly rare condition, but Ab-mediated disease due to transplacental crossing of maternal auto- or allo-Abs is a well-recognized and relatively frequent mechanism of organ dysfunction. This has been shown in the thyroid (congenital hypothyroidism), the blood (fetal and neonatal hemolytic disease and fetal and neonatal auto- and alloimmune thrombocytopenia), the neuromuscular junction (transient neonatal myasthenia gravis), the heart (congenital heart block due to SSA/Ro Abs), and other organs and tissues (132–137). We therefore hypothesize that neonatal GBS disease may be caused by yet-undiscovered neutralizing maternal auto-Abs or allo-Abs against components of the fetal and neonatal immune system that are non-redundant in conferring neonatal protection against GBS. The progressive decay of circulating maternal Abs in the infant plasma might then explain the decreasing incidence of infection over the first 3 months of life. Furthermore, the presence of pathogenic circulating Abs in the maternal blood would be consistent with the occurrence of mild disease in the mother (GBS-related urinary tract infection or mastitis) that is often associated with neonatal GBS disease, as well as with the well-documented higher risk of GBS-EOD in infants with a previous sibling with GBS disease (7). Finally the removal, with exchange transfusion, of pathogenic Abs from neonatal plasma could be an additional explanation to the efficacy of the procedure in infants with septic shock (138).

The proposed mechanism could in part explain neonatal GBS disease in full-term infants. Despite transplacental transfer of Abs is reduced at low gestational ages, allo- or autoimmune pathogenic maternal Abs have been demonstrated to be able to cause disease in the preterm infant or during gestation (71, 134). Therefore, the “maternal antibody” hypothesis could also explain some cases of neonatal GBS disease occurring in preterm infants.

### Testing the Hypotheses—Possible Study Methodologies

Previous studies that addressed the role of genetics in the susceptibility to neonatal infection focused on the associations between selected common single-nucleotide polymorphisms and infectious outcomes (139). A more integrated approach including genomics, transcriptomics, proteomics, and functional studies is required to uncover the precise molecular determinants of susceptibility to specific pathogens causing neonatal infections.

*Ad hoc* studies should be designed depending on the phenotype under investigation.

Multicenter GWASs may provide some insight into the pathogenesis of suspected multifactorial infections as, for instance, those occurring in preterm infants. GWAS are currently ongoing on neonatal cohorts (54).

Exome or genome sequencing studies have potential to uncover the cause of suspected monogenic disorders. Cases should be prioritized based on the clinical profile most suggestive of a monogenic etiology, including extreme severity, consanguinity, recurrence of infection, and familial presentation. Depending on the design of the study, analysis of the trio (proband and parents) and of the family or cohort studies should be carried out to uncover the individual, rare (<1% in the general population), and functionally deleterious genetic variants that best fit the most likely genetic model (*de novo*, autosomal dominant with complete or incomplete penetrance, autosomal recessive with mono- or biallelic mutations). This approach could shed light on the pathways that are non-redundant in neonatal protection against GBS.

Functional follow-up will be required to validate candidate variants and confirm their causative role. These studies should be designed to assess the integrity of the molecular pathways affected by the mutations and determine how they are relevant to the neonatal immune responses in primary cells and/or immortalized cell lines.

Laboratory experiments will be also needed to investigate the possible interfering effect of maternal plasma on the neonatal immune responses. The laboratory tests could include cytokine production assays, detailed analyses of RNA (transcriptome analysis) and protein expression in *ex vivo* samples (blood collected during sepsis), and *in vitro* experiments (stimulation of patient and control cells with different ligands, cell differentiation assays) in the presence of maternal or control plasma. Specific assays should be used for the detection of specific Abs in the maternal and in the perinatal plasma.

Ultimately, these experiments should aim at demonstrating a causative link between the molecular findings, the observed cellular phenotypes, and the patient’s clinical phenotype.

## Conclusion

Transient susceptibility to a narrow range of infections during the neonatal age may be explained by inborn errors of immunity, in the context of a relatively immature, non-redundant immune system. The early recognition of a PID as an essential contributing factor to a severe neonatal infection is clinically very relevant, as it may change the management and allow the referral of the patient to the clinical immunologist for specific follow-up and family counseling.

In parallel fields, the discovery of concurrent genetic and auto-/alloimmune mechanisms for several neonatal diseases has dramatically changed practice, as exemplified by the development of highly effective screening and diagnostic procedures for neonatal hemolysis, which reduced the incidence of fetal erythroblastosis and neonatal bilirubin encephalopathy by two orders of magnitude, from ~1/1,000 to ~1/100,000 live births (Table 2) (140). Similar observations can be made for congenital hypothyroidism and other common and rare neonatal diseases (Table 1).

**Table 2:** **Comparison of neonatal hemolytic disease and neonatal group B streptococcus (GBS) disease**.

	Neonatal hemolytic disease	Neonatal GBS disease
Physiological condition	Mild jaundice (~50% newborn infants)	GBS colonization (~10% of infants at birth; probably higher cumulative colonization rate during the first 3 months of life)

Disease	Life-threatening jaundice/kernicterus	Life-threatening infection

Incidence of disease in the absence of prevention	Estimated ~1/1,000	EOD: 1.8/1,000
		LOD: 0.3/1,000

Incidence after prevention	0.4–2.7/100,000	EOD: 0.3/1,000
		LOD: 0.3/1,000

Prenatal disease	Facultative: fetal anemia/erythroblastosis	Facultative: term/preterm premature rupture of membranes, chorioamnionitis, GBS-related stillbirth

Screening/early diagnosis	Highly effective: direct and indirect Coombs test/serial plasma bilirubin	Partially effective: universal screening of pregnant women for GBS/C-reactive protein, blood count, cultures after onset of infection

Prevention of life-threatening disease	Phototherapy	*Intrapartum* antibiotic prophylaxis

Treatment	Phototherapy; blood exchange	Antibiotics; intensive care; blood exchange

Molecular mechanisms	Known (red cells genetic defects, maternal AB0/Rh alloimmunization)	Unknown

*EOD, early-onset GBS disease; LOD, late-onset GBS disease*.

*Incidence is expressed as number per 1,000 (or 100,000) live births*.

Current prevention efforts, although invaluable for neonatal health, only had a limited impact on the global incidence of neonatal infections (9, 141) (Table 2). A more complete understanding of the mechanisms underlying the interindividual variability in the neonatal innate immune responses to pathogens is required to develop highly effective, pathogen-specific and individual-tailored preventive protocols.

## Author Contributions

AB conceived the manuscript; conducted the literature search; and drafted, edited, and approved the final version of the paper. MS participated in the discussion of ideas, helped with the writing, revised critically, and approved the final version of the manuscript. JF participated in the discussion of ideas, edited, revised critically, and approved the final version of the manuscript.

## Conflict of Interest Statement

The authors declare that the research was conducted in the absence of any commercial or financial relationships that could be construed as a potential conflict of interest.
